# Shoulder MRI features with clinical correlations in subacromial pain syndrome: a cross-sectional and prognostic study

**DOI:** 10.1186/s12891-017-1827-3

**Published:** 2017-11-21

**Authors:** Elisabeth Kvalvaag, Masoud Anvar, Anna Cecilia Karlberg, Jens Ivar Brox, Kaia Beck Engebretsen, Helene Lundgaard Soberg, Niels Gunnar Juel, Erik Bautz-Holter, Leiv Sandvik, Cecilie Roe

**Affiliations:** 1 Department of Physical Medicine and Rehabilitation, Oslo University Hospital HF, Ullevaal, Postboks 4956 Nydalen, 0424 Oslo, Norway; 2 University of Oslo, Medical Faculty, Boks 1072 Blindern, 0316 Oslo, Norway; 3 Department of Radiology, Oslo University Hospital HF, Ullevaal, Postboks 4956 Nydalen, 0424 Oslo, Norway; 4 University of Oslo, Faculty of Dentistry, Boks 1072 Blindern, 0316 Oslo, Norway

**Keywords:** MRI, Shoulder pain, Subacromial pain syndrome, Patient outcome, Prognosis

## Abstract

**Background:**

Previous studies on shoulder patients have suggested that the prevalence of rotator cuff or bursa abnormalities are weakly related to symptoms and that similar findings are often found in asymptomatic persons. In addition, it is largely unknown whether structural changes identified by magnetic resonance imaging (MRI) affect outcome after treatment for shoulder pain. The purpose of this study was therefore to evaluate the presence of structural changes on MRI in patients with subacromial pain syndrome and to determine to what extent these changes are associated with symptoms and predict outcome after treatment (evaluated by the Shoulder Pain and Disability Index (SPADI)).

**Methods:**

A prospective, observational assessment of a subset of shoulder patients who were included in a randomized study was performed. All participants had an MRI of the shoulder. An MRI total score for findings at the AC joint, subacromial bursa and rotator cuff was calculated. Multiple linear regression analysis was applied to examine the relationship between the MRI total score and the outcome measure at baseline and to examine to what extent the MRI total score was associated with the change in the SPADI score from baseline to the one year follow-up.

**Results:**

There was a weak, inverse association between the SPADI score at baseline and the MRI total score (β = −3.1, with 95% CI −5.9 to −0.34; *p* = 0.03), i.e. the SPADI score was higher for patients with a lower MRI total score. There was an association between the change in the SPADI score from baseline to the one year follow-up and the MRI total score (β = 8.1, 95% CI -12.3 to −3.8; *p* < 0.001), with a poorer outcome for patients with a higher MRI total score. Both tendinosis (*p* = 0.01) and bursitis (*p* = 0.04) were associated with a poorer outcome after one year.

**Conclusions:**

In this study, MRI findings were significantly associated with the change in the SPADI score from baseline and to one year follow-up, with a poorer outcome after treatment for the patients with higher MRI total score, tendinosis and bursitis on MRI.

**Trial registration:**

Clinicaltrials.gov no NCT01441830. September 28, 2011.

## Background

Shoulder pain is a common musculoskeletal problem that causes disability and pain for the patient, and sick leave expenses for the patient as well as for the society [1, 2]. The most frequent shoulder diagnosis is subacromial pain syndrome (also called shoulder impingement syndrome) [3]. It is important to establish valid diagnostic methods for these patients to potentially improve management and treatment of this syndrome.

Magnetic resonance imaging (MRI) has become a frequently used diagnostic tool for the evaluation of structural abnormalities in the shoulder. This includes the rotator cuff and the subdeltoid/subacromial bursa, and other structural abnormalities [4].

Previous studies have suggested that the prevalence of rotator cuff or bursa abnormalities is weakly related to symptoms and that similar findings are often found in asymptomatic persons. One study found changes (grade 1) in 80% of the supraspinatus tendons of asymptomatic baseball pitchers with no significant difference between the throwing and the non-throwing arm [5]. Another study found no significant differences for the prevalence of partial tears, AC joint degeneration or tendinopathy in symptomatic vs asymptomatic Ironman Triathletes [6]. One review reported that partial thickness tears of the rotator cuff were more common in asymptomatic volunteers than in individuals with painful shoulders [7]. Enhancement of the subacromial/subdeltoid bursa was not found to have any relationship to shoulder symptoms in symptomatic and asymptomatic rotator cuff tears in two studies [8, 9]. In contrast, another study found that subacromial bursal effusion was correlated to the reported severity of the shoulder disability in patients with subacromial impingement syndrome [10].

It is largely unknown whether structural changes identified by MRI affect the outcome of non-operative treatment for shoulder pain. One previous study reported no predictive value of rotator cuff tendon pathology or bursal exudation as detected via sonography or MRI on short term outcome after corticosteroid injection in patients with subacromial pain [11]. However, two other studies reported that patients with minor-grade (possibly reversible) MRI findings presented a more favorable course after conservative treatment compared to patients with more severe findings on MRI [12, 13].

Despite the weak correlation between clinical and radiologic findings, many patients with symptoms of subacromial pain syndrome are referred for an MRI, in Norway often already in primary health care. In addition, MRI findings (together with clinical examination) are often used as indications for surgery in this patient group.

The objective of this study was therefore to evaluate the presence of structural changes on MRI in patients with subacromial pain syndrome and to determine to what extent these changes are associated with symptoms and predict outcome after treatment (evaluated by the Shoulder Pain and Disability Index (SPADI)). We hypothesized that degenerative findings like tendinosis, bursitis, partial tears, AC joint osteoarthritis, calcification and acromial morphology detected on MRI in patients with subacromial pain syndrome are not related to symptoms (as determined by the Shoulder Pain and Disability Index (SPADI)) before and after exercise treatment.

## Methods

### Study design, setting and participants

A prospective observational assessment of patients with subacromial pain syndrome who were included in a randomized controlled trial was undertaken [14]. The trial (clinicaltrials.gov registry number NCT01441830) included 143 patients aged 25 to 70 years old with subacromial pain syndrome lasting at least 3 months. The patients were enrolled between January 2012 and April 2014 at the outpatient shoulder clinic at the Department of Physical Medicine and Rehabilitation, Oslo University Hospital. All patients provided written informed consent. A brief description of the original trial participants follows.

The inclusion criteria were pain on one of two isometric tests (abduction or external rotation), positive Hawkins-Kennedy impingement sign [15] and normal passive glenohumeral range of motion. The exclusion criteria were previous surgery on the affected shoulder, instability, rheumatoid arthritis, full thickness tear of the rotator cuff, cervical radiculopathy, infection, patients considered unable to fill out questionnaires or follow the treatment, contraindications for shock wave therapy (use of anticoagulant drugs, bleeding disorder, epilepsy, pregnancy or pacemaker), previous experience with shock wave therapy, injection of cortisone in the affected shoulder in the last six weeks and a SPADI score below 20.

Patients were randomly assigned to one of two treatment groups; rESWT and supervised exercises (*n* = 69) or sham rESWT and supervised exercises (*n* = 74). The rESWT/sham was conducted once a week in the first four weeks of the study. All patients were offered up to 20 supervised exercise sessions during the treatment period of 12 weeks, but for various reasons, many patients attended fewer sessions. The median number of sessions was 13 (range two to 20).

The primary study results after 24 weeks showed no significant differences in any outcome measures between the two treatment groups [14]. Of the 143 patients enrolled in the original trial, 115 had an available MRI of their painful shoulder and were included in the current study. For the patients with bilateral shoulder pain, the MRI of their most painful shoulder was included.

### Clinical evaluation and assessment

At baseline, the patients completed a self-assessment questionnaire that included demographic and clinical prognostic factors and the SPADI. A clinical examination was performed, and the active range of motion in abduction and the external rotation were recorded. The first author (EK) did all the clinical assessments. Range of motion in abduction was assessed with the patient sitting on a chair in front of a panel with the degrees from 0 to 180 printed on [16]. External rotation was measured sitting on the same chair, with the chair placed on a similar panel and the arm in neural position. The pain-provocing isometric strength test for abduction was performed with the shoulder in 30 degrees of abduction. The isometric external and internal rotation was performed in neutral position of the shoulder. The patients were asked whether the test was painful or not (yes/no) and the answer was recorded.

The SPADI is a self-assessment questionnaire with 13 questions divided into two subscales; one with five questions regarding shoulder pain and one with eight questions regarding shoulder function. Each question is scored on a visual analogue scale from 0 to 11. The total SPADI score ranges from 0 to 100, with 0 being no pain and disability and 100 being the worst possible pain and disability [17].

For this study, the patients came to a follow-up visit after one year.

### MRI protocol and review

The patients included in the randomized trial were referred for an MRI at the Radiologic Department at Oslo University Hospital (except for the patients with contraindications for MRI and/or claustrophobia). MRIs taken before referral to the shoulder clinic at the Department of Physical Medicine and Rehabilitation at Oslo University Hospital were accepted if the images had been obtained within the last three months.

When the data were analyzed, the following variables from the MRI examinations were used: Acromion type (I, II, III, IV), AC-joint osteoarthritis (yes/no), bursitis (yes/no), tendinosis in one or more tendons of the rotator cuff (yes/no), partial tear in one or more tendons of the rotator cuff (yes/no) and calcification in one of more tendons of the rotator cuff (yes/no).

**Fig. 1:**
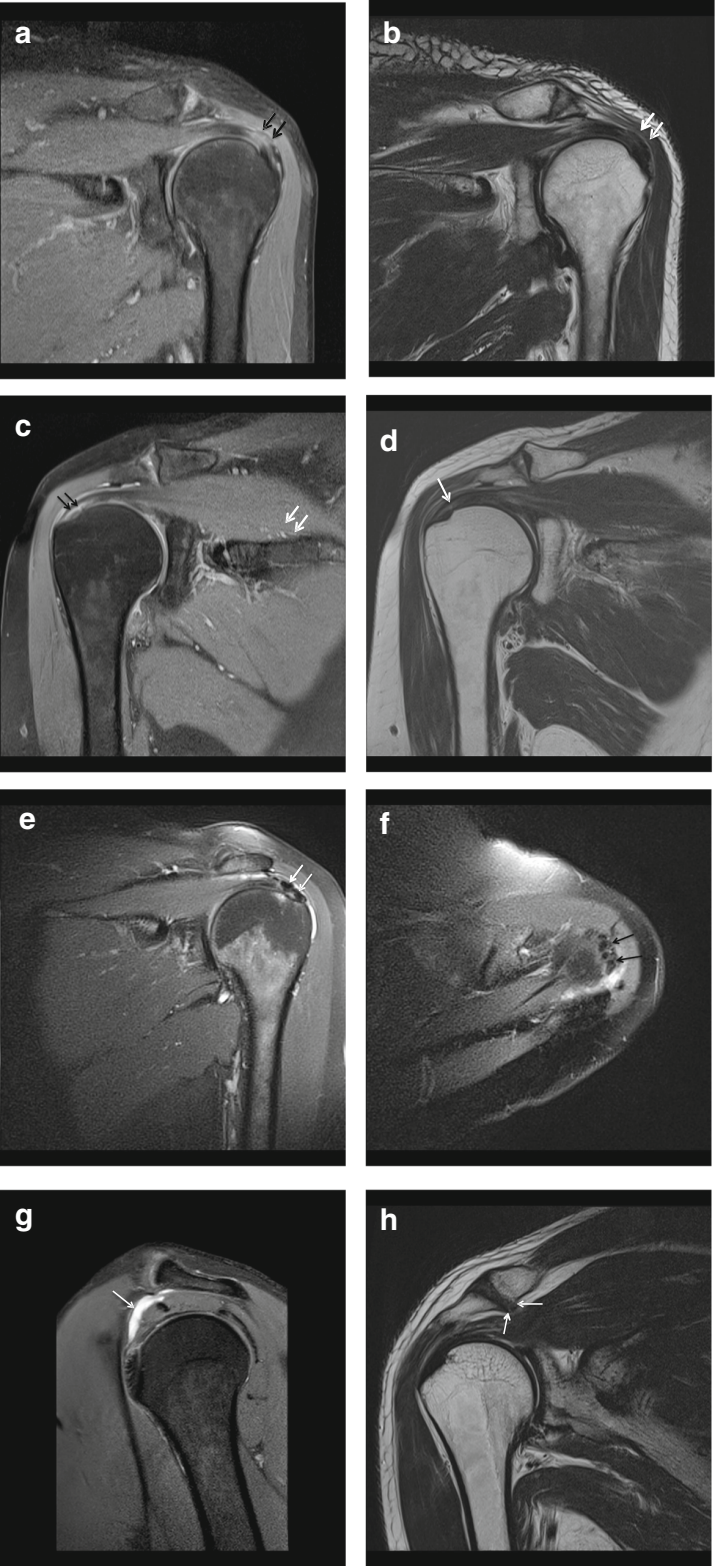
Oblique coronal PDFS-weighted (**a**, **c**, **e**, **g**), oblique coronal T2-weighted (**b**, **d**, **h**), and axial PDFS-weighted (**f**) MRI images in five different patients (**a**-**b**, **c**-**d**, **e**-**f**, **g**, **h**) illustrating typical MRI findings assessed in this study. **a** and **b** demonstrate tendinosis with thickening and increased intermediate signal within the supraspinatus tendon (black and white arrows). **c** and **d** demonstrate partial tears with signal abnormality in the undersurface extending to the intrasubstance in the supraspinatus tendon (black and white arrows). **e** and **f** demonstrate calcific tendinosis of the supraspinatus tendon with low density areas (white and black arrows) and edema in tendon and the subjacent bone. In addition there is slightly fluid in the subacromial/subdeltoid bursa. **g** demonstrates subacromial/subdeltoid bursitis with increased fluid and slightly thickening of the wall (white arrow). **h** demonstrates AC joint osteoarthritis with prominent undersurface osteophyte formation causing narrowing of the supraspinatus outlet (white arrows)

A previous study calculated an MRI total score for patients with low back pain and found no association between degenerative findings and pain and disability [18]. We therefore calculated a similar MRI total score for shoulder patients, using findings at the AC joint, subacromial bursa and rotator cuff and used this in the analyzis in addition to each of the individual findings. Each of the following findings contributed one point to the total MRI score: tendinosis in one or more tendons of the rotator cuff (yes/no), partial tear in one or more tendons of the rotator cuff (yes/no), calcification in one or more tendons in the rotator cuff (yes/no), bursitis (yes/no) and AC-joint osteoarthritis. See Fig. 1. Thus, the score ranged from 0 to 5 points. As there were only three patients with all five findings, they were included in the group of patients with four findings on MRI, and the maximum MRI total score could be four points.

#### Images

Sixty-two (53.9%) patients had a shoulder MRI from our hospital; the rest of the patients had an MRI from a private center taken shortly before they were included in the study. The MRI examinations included the following sequences: 111 (96.5%) had transverse PDFS images, 66 (57.5%) had sagittal T1 weighted images, 100 (86.9%) had sagittal T2-weighted images, 41 (35.7%) had coronal PD images, 85 (73.9%) had coronal T2-weighted images and 101 (87.8%) had coronal PDFS images. Four (3.5%) examinations were MRI arthrograms.

#### Image evaluation

Two radiologists who were blinded to clinical data and experienced in musculoskeletal MRI evaluated the images retrospectively with a clinical Picture Archiving and Communication System (PACS). The imaging reading software used was the Siemens Syngo Studio and Syngo Imaging VB36C. One observer had more than five years of experience, and the other had more than ten years. In a pilot study, the two observers evaluated ten shoulder MRIs to achieve a common understanding of the image evaluation criteria. The following criteria were used:

- AC joint osteoarthritis: Degenerative changes of the AC joint are defined by the presence of joint space narrowing, periarticular sclerosis or bone marrow edema, subchondral cyst formation, marginal osteophyte formation, joint effusion and capsular distention [19].
- Acromion morphology: The shape of the acromion as seen in sagittal oblique MR images was assessed. Four morphologies have been identified. In type I, the acromion is flat; in type II, the acromion is curved; in type III; the acromion is hooked; and in type IV, the acromion has a convex inferior contour [20].
- Subacromial-subdeltoid (SASD) bursitis: SASD bursitis is defined by thickening or distention of the bursa. This can been seen as a low intensity signal in T1 and increased signal intensity on T2, PD-weighted images or PDFS- weighted sequences [21].
- RC tendinosis: On MRI, tendinosis appears as swelling and an increased signal on low TE images, such as PDFS, STIR or T2 with fat suppression or an intermediate signal on T2- weighted images. However, the signal is not as bright as fluid [21, 22]. Calcific tendinosis is defined as the deposition of calcium within or around the rotator cuff tendons. It has low signal intensity on all pulse sequences, and there is usually edema within the tendon and occasionally within subjacent bone [21].
- RC Partial-Thickness Tears: A partial rotator cuff tear involves only a portion of the tendon. Partial tears are seen as a focal fluid signal within the tendon, without complete extension from the bursal to articular surface. If a chronic partial tear has started to develop granulation tissue, the signal may be somewhat hypointense to fluid. Partial tear subtypes include articular-sided, bursal-sided, or intrasubstance tears and include both delaminating tears as well as focal tears confined within the footprint of the tendon [21].

### Statistical analysis

Statistical analysis was performed with SPSS software (IBM SPSS for Windows, version 23, Chicago IL, USA). The data were analyzed by an independent statistician not involved in the radiologic or clinical data collection. Multiple linear regression analysis was carried out with the SPADI score at baseline and the change in the SPADI score from baseline to the one year follow-up as dependent variables and with the MRI total score as a covariate. We adjusted for age, gender, education, work status and emotional distress (HSCL-25). The multicollinearity, residuals and influential data point checks showed that the assumptions of the regression models were not violated (Cook distance <0.1, Centered Leverage value <0.2). Analysis with treatment arm as an additional covariate was also run without influencing the results. All analyses were adjusted for the baseline value of the dependent variable. The same analyses were performed with the individual dichotomized MRI findings as covariates, with separate analyses for each MRI finding. The variance inflation factor (VIF) was checked, and was low for all variables. All analyses were also run separately in each treatment arm providing similar results as for the pooled analysis.

To assess the robustness of the results, we also did multiple linear regression analysis with all individual MRI findings in the same analysis, and manual backwards selection until all remaining variables had a *p* value of below 0.05. SPADI baseline, age, gender, education, work status and emotional distress were kept in the model.

## Results

### Patient characteristics

Of the 115 patients included in this study, there were 62 women and 53 men. The mean age was 47 years. All patients had MRI and baseline data. Baseline data and comparisons with the 143 patients included in the original study are described in Table 1.

**Table 1:** Demographic and clinical factors at baseline. Values are numbers (percentages) unless stated otherwise

Variable	Population with MRI (*n* = 115)	Original population (*n* = 143)
Age (years), mean (SD)	47.0 (10.2)	46.7 (10.5)
Education		
≤ 12 years at school	10 (8.7)	13 (9.1)
University/college	60 (52.2)	74 (51.7)
Full- or part time work	80 (69.6)	130 (65.2)
Female sex	62 (54.4)	78 (54.4)
Emotional distress (1–4). Mean (SD)	1.6 (0.5)	1.6 (0.5)
SPADI baseline. Mean (SD)	52.4 (17.0)	51.9 (17.0)

One hundred and four of the 115 patients completed the study and had available SPADI scores after one year. The mean SPADI score at baseline was 52.4 (SD 17.0). There were 28 (24.3%) patients with acromion type I, 78 (67.8%) patients with acromion type II, four (3.5%) patients with acromion type III and five (4.3%) patients with acromion type IV. There were 28 patients (24.3%) with calcification in the rotator cuff, 85 (73.9%) with tendinosis, 40 (34.8%) with a partial tear in the rotator cuff, 65 (56.4%) with subacromial bursal effusion and 82 (71.3%) with AC joint osteoarthritis. There were eight patients with none of these structural findings on MRI, and three patients with all, i.e. calcification, tendinosis, subacromial bursal effusion, AC joint osteoarthritis and partial tear in the rotator cuff.

After one year, the mean SPADI score was 29.7 (SD 26.1), and the mean change in the SPADI score from baseline to the one year follow-up was 22.1 (SD 25.7).

### Clinical examination

The only variable from the clinical examination at baseline associated with a higher SPADI score at baseline was the active range of motion in abduction. For each 11 degree increase in AROM abduction, the SPADI score would decrease by 1.7 points (*p* < 0.001, 95% confidence interval (CI) -1.0 to −0.30). No recorded variables from the clinical examination had any predictive value when evaluating the change in the SPADI score during one year follow-up.

### MRI findings vs. SPADI baseline

The MRI total score was weakly correlated to the SPADI score at baseline (β = −3.1, with 95% CI −5.9 to −0.34; *p* = 0.03, R^2^ 1.9%), as seen in Table 2. There were no significant correlations between the SPADI score at baseline and individual structural changes on MRI, except for calcification in the rotator cuff (β = −8.2, with 95% CI −15.4 to −1.1; *p* = 0.03, R^2^ 4.4%) (Table 2). For both the MRI total score and the individual calcification in the rotator cuff the correlation was inverse, i.e., the patients with calcification or high MRI total score had a lower SPADI score at baseline than the patients without calcification or a low MRI total score.

**Table 2:** Analysis of the relationship between baseline SPADI and the MRI total score and individual structural changes at MRI, calculated by multiple regression analysis, adjusting for age, gender, work status, education and emotional distress

	Structural change present	SPADI baseline^a^	Diff SPADI baseline (β)	*P* value	95% confidence interval
MRI total score	–	–	−3.1	0.03	−5.9 to −0.34
Bursitis	YES	51.5 (17.1)	−3.1	0.35	−9.6 to 3.4
	NO	53.9 (16.8)			
Tendinosis in rotator cuff	YES	52.8 (16.8)	2.1	0.58	−5.3 to 9.4
	NO	51.2 (17.8)			
Calcification in rotator cuff	YES	46.1 (12.8)	−8.2	0.03	−15.4 to −1.1
	NO	54.4 (17.7)			
Partial tear in rotator cuff	YES	50.1 (15.0)	−3.6	0.33	−10.9 to 3.7
	NO	53.6 (17.9)			
AC joint osteoarthritis	YES	51.8 (17.8)	−3.5	0.35	−11.1 to 4.0
	NO	53.9 (14.8)			

^a^Values are mean (SD)

We also did analysis with all the individual MRI findings in a single model and manual backwards selection. The only remaining variable with a *p* value below 0.05 in this analysis was calcification in the rotator cuff (with the same result as presented above).

### MRI findings vs. change in the SPADI score after one year

**Fig. 2:**
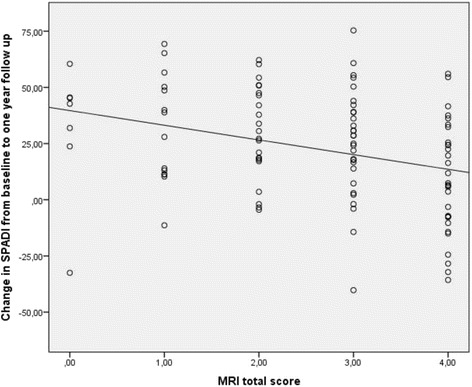
Change in SPADI score from baseline to one year follow up for the patients with zero to four points on the MRI total score, from multiple linear regression analysis

After one year, the MRI total score was significantly correlated to the change in the SPADI score, with an 8.1 point decrease in the change in the SPADI score for each structural change that was detected at MRI (95% CI -12.3 to −3.8; *p* < 0.001, R^2^ 8.8%), see Table 3 and Fig. 2.

**Table 3:** Analysis of the relationship between the change in SPADI over one year and the MRI total score and individual structural changes at MRI, calculated by multiple regression analysis, adjusting for baseline SPADI, age, gender, work status, education and emotional distress

	Structural change present	Change in SPADI from baseline to one year follow up^a^	Diff change SPADI (β)	*P* value	95% confidence interval
MRI total score	–	–	−8.1	< 0.001	−12.3 to −3.8
Bursitis	YES	18.4 (26.0)	10.2	0.04	0.3 to 20.1
	NO	28.0 (24.5)			
Tendinosis in rotator cuff	YES	18.8 (25.7)	14.1	0.01	3.3 to 25.0
	NO	31.1 (23.9)			
Calcification in rotator cuff	YES	13.9 (27.4)	7.0	0.22	−4.2 to 18.1
	NO	24.9 (24.7)			
Partial tear in rotator cuff	YES	20.2 (24.2)	2.4	0.69	−9.5 to 14.3
	NO	23.1 (26.6)			
AC joint osteoarthritis	YES	19.2 (24.9)	10.8	0.08	−1.1 to 22.6
	NO	29.8 (26.9)			

^a^Values are mean (SD)

Of the individual structural changes, tendinosis (β = 14.1, with 95% CI 3.3 to 25.0; *p* = 0.01, R^2^ 4.5%) in the rotator cuff and bursitis (β = 10.2, with 95% CI 0.3 to 20.1; *p* = 0.04, R^2^ 3.0%) at baseline was associated with a poorer outcome. There was also a tendency towards a poorer outcome for the patients with AC joint osteoarthritis and calcification in the rotator cuff at baseline, but this difference was not statistically significant (Table 3). We also did analysis with all the individual MRI findings in a single model and manual backwards selection. The only remaining variable with a *p* value below 0.05 in this analysis was tendinosis in the rotator cuff (with the same result as presented above).

There was no difference in the mean change in the SPADI score after one year for the patients with a flat/upward acromion (type I and IV) vs. the patients with a hooked acromion (type II and III); β = −1,1, 95% CI -12.2 to 9.9, *p* = 0.84.

## Discussion

The main finding of the current study was that a higher MRI total score (more structural findings) predicted a poorer outcome after one year. Of the individual structural findings, tendinosis in one or more tendons of the rotator cuff and bursitis predicted a poorer outcome.

Few previous studies have evaluated whether structural changes detected on MRI influence prognosis after conservative treatment in this patient group. Ekeberg et al. found no contribution to their predictive model on short term outcome (six weeks) after corticosteroid injection when considering rotator cuff or bursal abnormalities detected on MRI and sonography [11]. However, their study had a short follow-up and no standardized description of the MRIs. Ertan et al. found that patients with “minor-grade” MRI findings presented a more favorable course in the long term, which supports our findings, even though their study used a different MRI classification than ours [12]. Hambly et al. reported similar findings as Ertan et al. and used even another MRI classification system [13]. Ketola et al. found that patients with AC joint degeneration had more pain five years after surgery for shoulder impingement syndrome [23]. However, AC joint degeneration is very common, and there is poor evidence for the effectiveness of surgery [24–26].

In contrast to our study, some previous studies have reported a poorer outcome after conservative treatment in patients with a hooked acromion (type II and III), [27, 28] while other studies have called into question the relevance of the shape of acromion, and reported low interobserver reliability for assessing acromial shape [29–31].

At baseline, we found a weak inverse correlation between the SPADI score and the MRI total score. The MRI total score explained only 1.9% of the variance in baseline SPADI and the observed weak association has no clinical significance. We speculate however that one possible explanation may be that the general practitioners refer more patients with several structural changes on MRI, including patients with a low symptom burden. Previous studies have shown conflicting results regarding the association between structural changes detected on MRI and symptoms, but an inverse relation has not been reported. Reuter et al. found no significant differences in the prevalence of partial tears, AC joint degeneration or tendinosis in symptomatic vs. asymptomatic Ironman Triathletes [6]. One review reported that partial thickness tears of the rotator cuff were more common in asymptomatic volunteers than in individuals with painful shoulders [7]. Enhancement of the subacromial/subdeltoid bursa was not found to have any relationship to shoulder symptoms in individuals with symptomatic and asymptomatic rotator cuff tears in two studies, [8, 9] but in contrast, Ardic et al. reported that subacromial bursal effusion was correlated to the reported severity of shoulder disability in a small cohort including patients with subacromial impingement syndrome [10].

Results in the present study suggest inferior clinical results after one year in patients with more degenerative changes of the subacromial structures detected on MRI (in particular tendinosis and bursitis). However, the variance explained by MRI findings was low, the total score explained less than 10% and tendinosis or bursistis less than 5%. Still, future studies should evaluate tendon morphology before and after exercises and examine the relation between exercise type, load, intensity and duration. We may hypothesize that patients with more degenerative findings may benefit from slower progression and longer duration and that this was not obtained in the present study. Compliance with exercise therapy varied as suggested by the range of sessions reported and reasons for lack of compliance may include low motivation and difficulties in fitting exercise sessions with the work schedule.

The main strengths of the present study are that we included a large number of patients with subacromial pain syndrome diagnosed according to prespecified criteria. Two experienced radiologists that were blinded for clinical data reexamined all MRIs in conference, and conclusions were reached by consensus. We used the SPADI score, which is a reliable and validated shoulder questionnaire for the evaluation of the patient’s pain and function [32].

The major limitation of this study is that the MRI total score used in this study is not previously validated. Second, the patients were not blinded to the result of their MRI scan. We cannot exclude that the interpretation of MRI findings, how patients are informed about the findings, and how they finally attribute findings to their complaints, may influence the observed association between MRI findings and self-reported pain and disability after one year. It is not unlikely that patients with a higher number of more pronounced degenerative findings to a larger extent are informed that pain is caused by the observed MRI findings as compared to those with fewer degenerative findings. Ideally, in studies that evaluate the association between degenerative findings and symptoms, patients should be blinded to the description of MRI during follow-up.

In addition, different MRI scanners were used for the imaging. Moreover, the structural changes detected on MRI have not been confirmed by surgery. Previous studies have reported variable results regarding the diagnostic accuracy of shoulder MRI, especially when evaluating partial thickness tears and tendinosis, [33, 34] but we used a consensus between two experienced radiologists to improve the validity of the MRI evaluation. Also, we did not obtain MRIs at one year follow-up to assess the evaluation between an eventual reversal of morphology and changes in symptoms.

In addition, the high prevalence of degenerative findings in the asymptomatic population are consistently reported for the shoulder [5–7] and other musculoskeletal locations [35–37]. Thus, degenerative findings similar to those observed in the present study are normally found in persons with no pain or disability [38].

Moreover, some of the confidence intervals were quite wide, suggesting more uncertainty and the sample size should have been larger. Further studies with more patients are needed to confirm the results.

Accordingly, the results from the present study should be interpreted with caution.

## Conclusions

In this study, MRI findings were significantly associated with the change in the SPADI score from baseline and to one year follow-up, with a poorer outcome after treatment for the patients with higher MRI total score, tendinosis and bursitis on MRI.

## Abbreviations

AC joint: Acromio-clavicular joint
MRI: Magnetic Resonance Imaging
RESWT: Radial Extracorporeal Shock Wave Therapy
SPADI: The Shoulder Pain and Disability Index
